# Diagnostic accuracy of contrast-enhanced mammography in evaluating breast cancer response to neoadjuvant therapy: a systematic review and meta-analysis

**DOI:** 10.1007/s11547-026-02215-y

**Published:** 2026-05-21

**Authors:** Francesca Galati, Fatemeh Shakki Katouli, Roberto Maroncelli, Gloria Barcaroli, Narges Azizi, Alessandra Spagnoli, Elena Fabrizi, Federica Pediconi

**Affiliations:** 1 Department of Radiological, Oncological and Pathological Sciences, Sapienza - University of Rome, Piazzale Aldo Moro 5, 00185 Rome, Italy; 2 Department of Radiology, Shariati Hospital, Tehran University of Medical Sciences, Tehran, Iran; 3 Department of Experimental Medicine, Sapienza - University of Rome, Viale Regina Elena, 324, 00161 Rome, Italy; 4 Advanced Diagnostic and Interventional Radiology (ADIR), Tehran University of Medical Sciences, Tehran, Iran; 5 Department of Public Health and Infectious Diseases, Sapienza-University of Rome, 00161 Rome, Italy; 6 Department of Political Sciences, University of Teramo, 64100 Teramo, Italy

**Keywords:** Contrast-enhanced mammography, Neoadjuvant therapy, Breast cancer, Pathological complete response, Diagnostic accuracy

## Abstract

**Purpose:**

Neoadjuvant therapy (NAT) in breast cancer enables tumor downstaging and response evaluation. Although magnetic resonance imaging (MRI) is the gold standard, its limitations have prompted growing interest in alternatives such as contrast-enhanced mammography (CEM).

**Methods:**

This review was conducted according to PRISMA guidelines. We evaluated CEM accuracy in predicting pathological complete response after NAT, using surgery as reference standard. A comprehensive search was conducted in PubMed/Medline, Embase, Cochrane, and Web of Science. Two reviewers screened studies, extracted data, and evaluated risk of bias with QUADAS-2 tool. A bivariate random-effects model estimated diagnostic odds ratio (DOR), sensitivity, specificity, and likelihood ratios. Summary receiver operating characteristic (sROC) curves were generated, and heterogeneity was quantified using Higgins’ I^2^. Publication bias was assessed via Deek’s funnel plot.

**Results:**

Fifteen studies, including 793 patients, met inclusion criteria. The pooled analysis showed a DOR of 9.30 (95% CI 4.15–20.83) with substantial heterogeneity (I^2^ = 72.30%). Pooled sensitivity and specificity were 74% (95% CI 66%–81%) and 82% (95% CI 67%–91%), respectively, with an sROC AUC of 0.83 (95%, CI 0.67–0.92). Excluding the study contributing most to heterogeneity improved the DOR to 10.28 (95% CI 6.35–16.65) and reduced I^2^ to 19.4% (*p* = 0.242).

**Conclusions:**

CEM demonstrates good diagnostic accuracy for assessing NAT response, performing comparably to MRI.

*Trial registration* This systematic review is registered in PROSPERO under the registration number CRD420251003406.

## Background

Neoadjuvant therapy (NAT) has become a cornerstone in breast cancer management, initially designed to downstage tumors and facilitate breast-conserving surgery in locally advanced and non-operable cases, such as inflammatory breast carcinoma. Over time, its indications have expanded to include early-stage breast cancer, where it enables in vivo assessment of tumor response, thereby assisting in prognostic evaluation and treatment planning. Beyond improving resectability, NAT has been associated with better cosmetic outcomes and enhanced overall survival [1–3].

The assessment of treatment response to NAT occurs in two phases: (1) preoperative evaluation, which integrates clinical examination and imaging, and (2) postoperative evaluation, where histopathology remains the gold standard for confirming pathological complete response (pCR) [4]. Imaging plays a crucial role in monitoring NAT response, with the Response Evaluation Criteria in Solid Tumors (RECIST 1.1) being the most widely accepted classification system, categorizing response as complete, partial, stable disease, or progressive disease [5]. The most commonly employed imaging modalities include mammography, ultrasound, and breast magnetic resonance imaging (MRI) [6]. Among these, MRI is considered the reference standard due to the ability to provide functional (diffusivity, ADC mapping), morphological (tumor size and shrinkage patterns), and kinetic (vascularization) information [5, 7]. However, MRI high cost, limited accessibility, and suboptimal performance in evaluating microcalcifications limit a widespread implementation [8].

In this context, contrast-enhanced mammography (CEM) has emerged as a promising alternative, combining the anatomical resolution of mammography with functional contrast-enhanced imaging similar to MRI, thus enabling direct visualization of tumor angiogenesis [9]. Compared to MRI, CEM offers key advantages, including greater availability, lower cost, shorter acquisition time, and better patient tolerance, especially for individuals with claustrophobia or MRI-incompatible implants [10, 11]. Recent studies suggest that CEM achieves diagnostic accuracy comparable to MRI, with reported sensitivity ranging from 89 to 98% and specificity between 58 and 84% [8, 12, 13]. Moreover, some studies indicate that CEM may reliably predict pCR, with sensitivity reaching 100% and specificity 84% [14, 15].

Despite these promising results, further validation is required to define the role of CEM in routine clinical practice and establish CEM performance as a substitute for MRI in treatment response monitoring. Therefore, this systematic review and meta-analysis aims to determine the diagnostic accuracy of CEM in assessing pCR after NAT, using surgical histopathology as the reference standard.

## Materials and method

### Study design

This systematic review was conducted following the Preferred Reporting Items for Systematic Reviews and Meta-Analyses (PRISMA) guidelines and was registered in PROSPERO under the identifier CRD420251003406 (https://www.crd.york.ac.uk/PROSPERO/view/CRD420251003406) [16, 17].

The study protocol was developed to ensure methodological rigor and transparency, outlining predefined objectives, eligibility criteria, data extraction procedures, and statistical methods for synthesis.

The study adhered to the original PROSPERO protocol, with no deviations from the registered methodology.

### Search strategy

A comprehensive literature search was performed across PubMed/MEDLINE, Embase, Cochrane Library, and Web of Science, including studies published up to October 10, 2025, to identify studies evaluating the diagnostic accuracy of CEM in assessing pCR after NAT. The search strategy incorporated a combination of MeSH terms and free-text keywords, including *“breast cancer,” “neoadjuvant therapy,” “RECIST,” “contrast-enhanced mammography,”* and *“diagnostic accuracy.”*

No restrictions were applied regarding publication date, but only peer-reviewed original research articles published in English were considered. The search process was conducted independently by two reviewers (FG, FSK), ensuring consistency and reproducibility. To further enhance completeness, reference lists of all included studies were manually screened for additional relevant publications.

### Study selection

Following the removal of duplicate records, two independent reviewers (FG, FSK) screened the titles and abstracts of all retrieved articles to identify potentially eligible studies. Full-text evaluations were conducted for articles that met the preliminary inclusion criteria, with any discrepancies resolved by consensus or through consultation with a third reviewer (FP).

The inclusion criteria were defined to ensure methodological robustness and clinical relevance. Eligible studies included randomized controlled trials, cohort studies, case–control studies, or case series with a minimum of four cases, provided they assessed the diagnostic accuracy of CEM following NAT.

Only studies in which image interpretation was performed by human readers and histopathology served as the reference standard were considered. Furthermore, studies were required to report sufficient data to construct a 2 × 2 contingency table**,** allowing for the calculation of sensitivity, specificity, and other diagnostic accuracy measures.

Exclusion criteria were applied to maintain the integrity of the review. Studies were excluded if they presented inconsistent or insufficient data**,** focused on animal or ex vivo models**,** were duplicate publications, secondary analyses of previously published data, or if they were published in languages other than English. The selection process was conducted in accordance with PRISMA guidelines, ensuring transparency and reproducibility in study inclusion.

### Data extraction and quality assessment

Data extraction was independently conducted by two reviewers using a standardized form to ensure consistency and completeness. Extracted information included publication year, study design, sample size, patient characteristics, and diagnostic accuracy measures, specifically true positives, false positives, true negatives, and false negatives.

In studies that included a comparison between CEM and MRI, only data related to the diagnostic performance of CEM were extracted and analyzed, without directly evaluating the comparison between the two modalities.

All included studies applied RECIST 1.1 criteria for response evaluation and defined pathological complete response (pCR) consistently as ypT0/is N0.

True negatives were defined as cases with no abnormal enhancement on CEM and confirmation of pCR on histopathology, whereas true positives were cases where CEM showed enhancement, and residual disease was identified on pathology. Any discrepancies in data extraction were resolved by consensus, with the involvement of a third reviewer (FP) when necessary.

The risk of bias was assessed using the Quality Assessment of Diagnostic Accuracy Studies-2 (QUADAS-2) tool, evaluating four key domains: patient selection, index test, reference standard, and flow/timing [18]. Each domain was independently reviewed by the two assessors, ensuring methodological rigor and minimizing subjectivity in the evaluation process. Disagreements were resolved through discussion to reach a consensus.

The final assessment of bias risk was systematically visualized using IBM SPSS Statistics (version 28) to generate statistical and graphical representations, enhancing transparency in reporting.

### Statistical analysis

Diagnostic performance was analyzed using forest plots and summary receiver operating characteristic (sROC) curves, estimating sensitivity and specificity with 95% confidence intervals (CI). Area under the curve (AUC) values were categorized as excellent (> 0.9), good (0.8–0.9), fair (0.7–0.8), poor (0.6–0.7), or failed discrimination (< 0.6) [19]. A bivariate random-effects model was applied to compute diagnostic odds ratio (DOR), positive likelihood ratio (LR +), and negative likelihood ratio (LR−).

To ensure methodological consistency, studies reporting multiple diagnostic performance metrics for different image interpretation approaches were analyzed separately as independent study components within the meta-analysis framework.

Heterogeneity across studies was assessed using Higgins’ I^2^ statistic, quantifying the proportion of variability due to heterogeneity rather than chance. An I^2^ value > 50% was considered substantial heterogeneity. Additionally, Kendall’s Tau (τ) was used to measure consistency among studies. The DOR metric, which integrates sensitivity and specificity, was also used for heterogeneity evaluation (DOR = sensitivity/(1 − sensitivity) ÷ (1 − specificity)/specificity) [20]. Publication bias was examined using Deek’s funnel plot asymmetry test, with *P* < 0.05 indicating significant bias [21]. Predictive values, including positive predictive value (PPV) and negative predictive value (NPV), were estimated based on a literature-reported pCR prevalence of 31.7%, and a Fagan nomogram was constructed to assess post-test probabilities [22, 23]. A leave-one-out sensitivity analysis was conducted to evaluate the robustness of findings. All statistical analyses were performed using Stata (version 14.2) and the Midas Package (StataCorp, College Station, TX, USA).

## Results

### Study selection

**Fig. 1:**
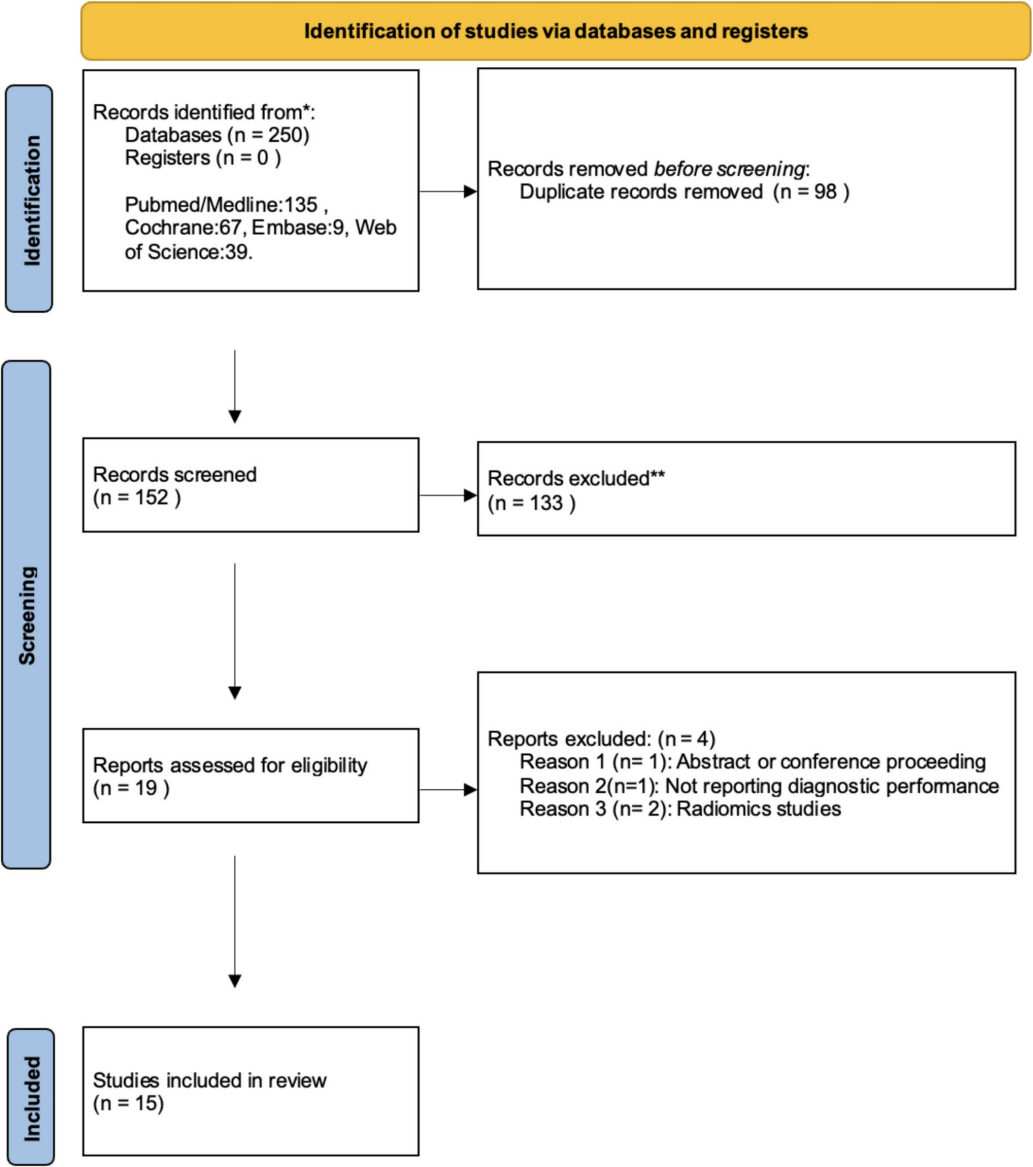
The flowchart represents the process of study selection

The study selection process is illustrated in the PRISMA flowchart (Fig. 1).

The initial literature search across electronic databases identified 250 articles. After removing 98 duplicates, 152 unique studies were screened by title and abstract, leading to the exclusion of 133 studies that did not meet the inclusion criteria. Full-text review was performed on the remaining 19 studies, of which 4 were further excluded due to insufficient data for diagnostic accuracy assessment or lack of histopathologic confirmation. Ultimately, 15 studies published between 2017 and 2024 were included in the systematic review.

One of the included studies presented two distinct sets of CEM diagnostic performance metrics, corresponding to different image interpretation approaches.

### Study characteristics

The key characteristics of the 15 included studies are summarized in Table 1.

**Table 1:** Characteristics of 15 included studies

First author, year of publication	Country	Study design	Patients	Reference standard	TP	FP	TN	FN
Iotti, 2017 [24]	Italy	Prospective	46	Surgical pathology	32	0	8	6
ElSaid, 2017 [25]	Egypt	Prospective	21	Surgical pathology	4	1	10	6
Barra, 2017 [26]	Brazil	Retrospective	8	Surgical pathology	5	0	2	1
Barra, 2018 [27]	Brazil	Prospective	33	Surgical pathology	19	5	8	6
Patel, 2018 [28]	USA	Retrospective	65	Surgical pathology	30	1	19	15
Moustafa, 2019 [29]	Egypt	Prospective	42	Surgical pathology	2	5	34	1
Steinhof-Radwanska, 2021 [30]	Poland	Retrospective	63	Surgical pathology	30	3	18	12
Steinhof-Radwanska, 2021 [30]	Poland	Retrospective	63	Surgical pathology	30	3	18	12
Iotti, 2021 [31]	Italy	Retrospective	36	Surgical pathology	26	1	2	7
Bernardi, 2022 [32]	Italy	Prospective	51	Surgical pathology	29	3	13	6
Canteros, 2022 [33]	Chile	Retrospective	48	Surgical pathology	9	15	5	19
Hogan Molly, 2023 [34]	USA	Prospective	110	Surgical pathology	67	16	16	16
Savaridas, 2023 [14]	UK	Prospective	14	Surgical pathology	4	0	9	3
Vidali, 2024 [35]	Italy	Retrospective	174	Surgical pathology	82	27	43	22
Mariscal Martínez, 2024 [36]	Spain	Prospective	42	Surgical pathology	25	1	13	3

Among these, 8 were prospective studies and 7 were retrospective studies, conducted by 12 independent research groups across 8 different countries. Collectively, these studies reported data from 793 patients who underwent CEM after completing NAT to assess treatment response.

All studies were single-center investigations, and in each case, histopathology served as the reference standard for determining pCR. The study populations varied in terms of tumor subtypes, NAT regimens, and imaging protocols, although all studies assessed CEM performance in detecting residual disease or confirming pCR.

### Quality assessment

The quality of the included studies was evaluated using the QUADAS-2 tool. The results of the assessment of the studies included in this systematic review are reported below. Low risk of bias was identified in 11/15 assessments (73.3%) for the *Patient Selection* domain, 9/15 (60%) for the *Index Test*, 13/15 (86.6%) for the *Reference Standard*, and 14/15 (93.3%) for *Flow and Timing*. High risk of bias was most frequently observed in the *Patient Selection* (3/15, 20%) and *Index Test* (3/15, 20%) domains. Unclear risk was attributed in several assessments due to insufficient information, particularly regarding blinding procedures and reporting of diagnostic thresholds.

**Fig. 2:**
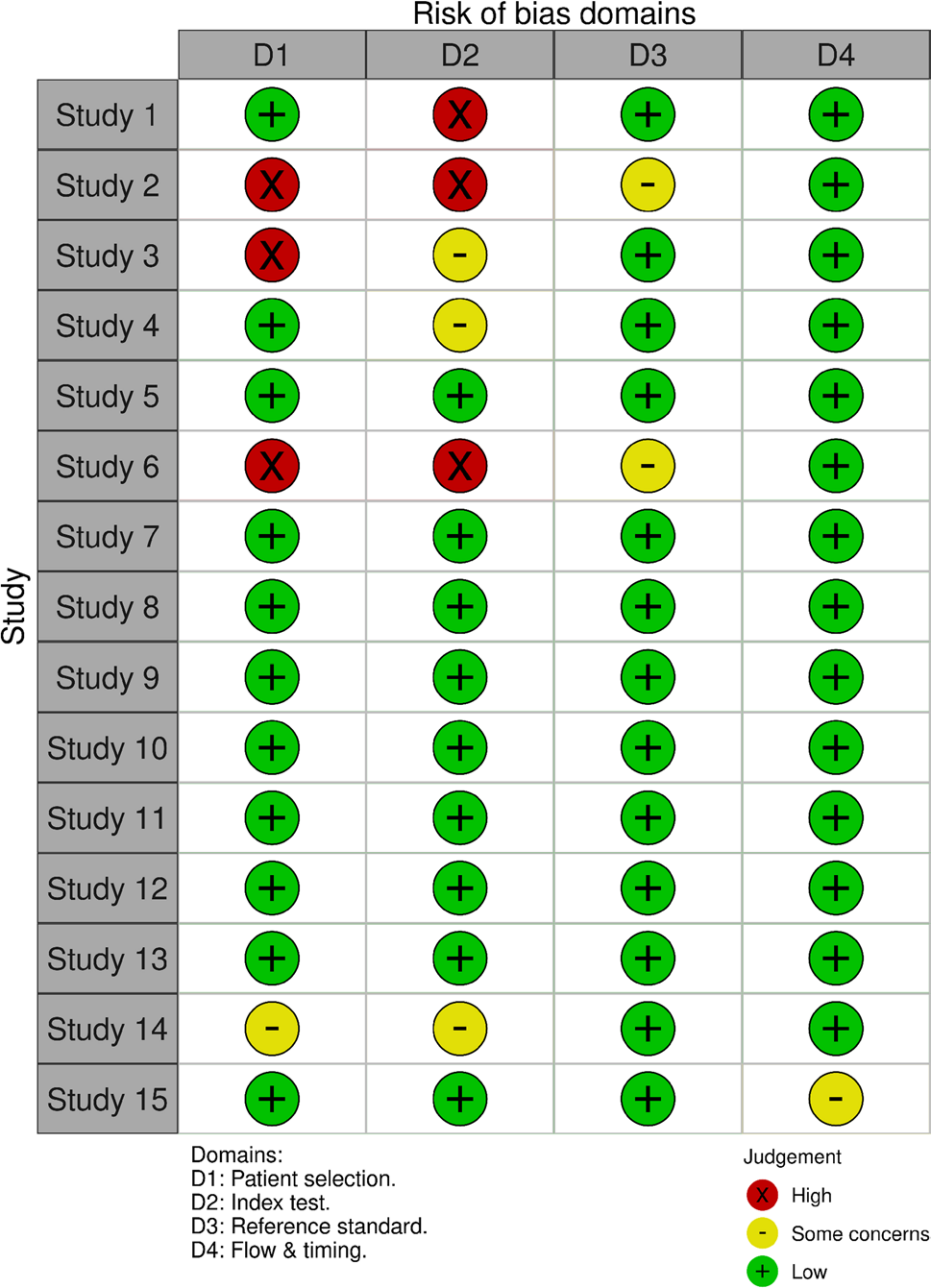
Risk of bias assessment of the included studies using the QUADAS-2 tool. Each row represents an individual study (15 total evaluations), and each column corresponds to a QUADAS-2 domain: D1 (Patient selection), D2 (Index test), D3 (Reference standard), and D4 (Flow and timing). Green indicates low risk of bias, yellow indicates unclear risk, and red indicates high risk. Most studies showed low risk of bias across all domains, with the highest concerns observed in the *Patient selection* and *Index test* domains

A graphical summary of the risk of bias is presented in supplemental material (Fig. 2).

### Diagnostic accuracy of CEM compared to surgical histopathology

**Fig. 3:**
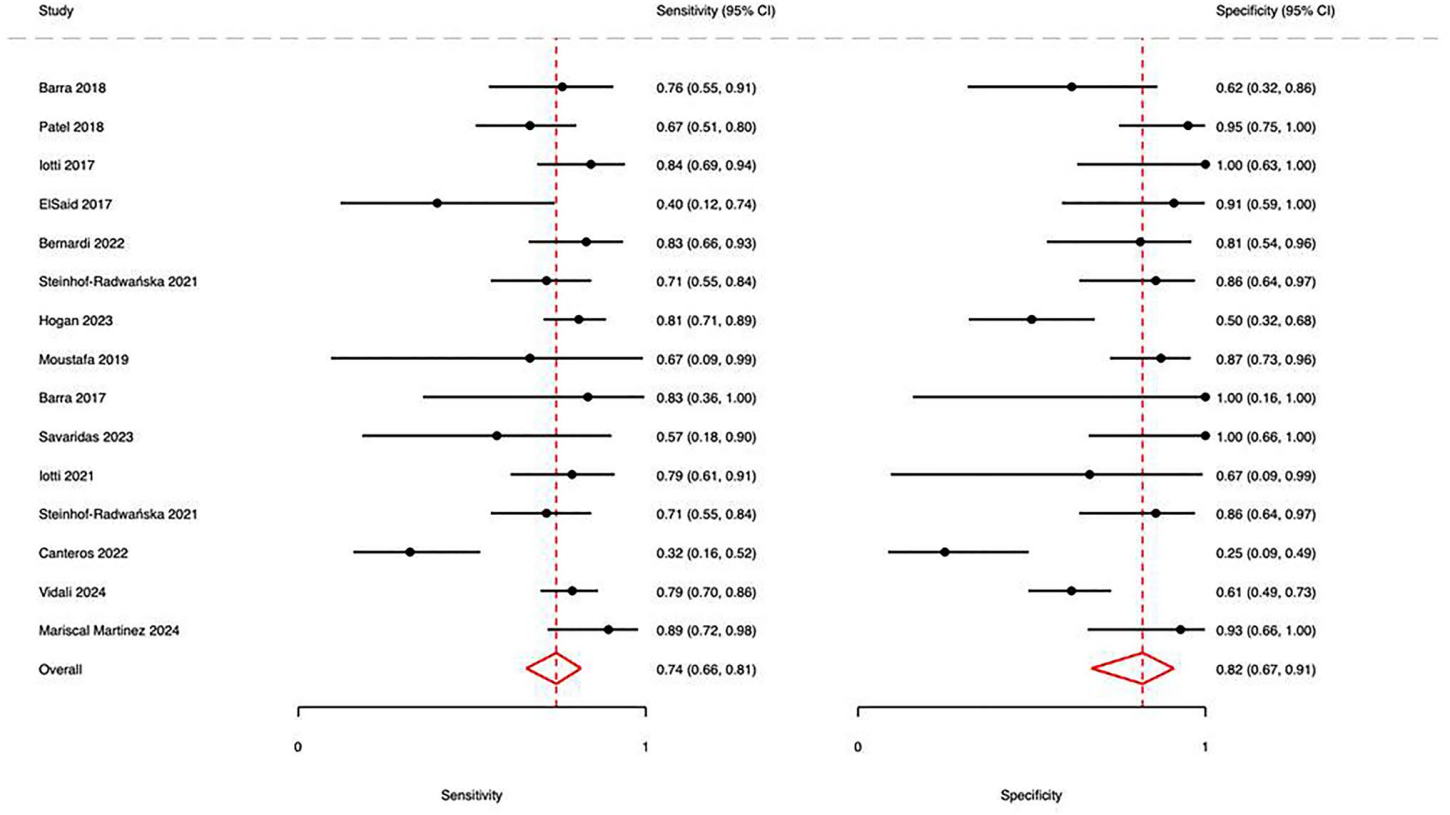
Diagnostic odds ratio (DOR) of contrast-enhanced mammography (CEM) in predicting response to neoadjuvant therapy (NAT) in breast cancer. Circles and lines represent point estimates and 95% confidence intervals, respectively. The diamond represents the pooled DOR and their 95% confidence intervals

The pooled diagnostic accuracy analysis demonstrated a diagnostic odds ratio (DOR) of 9.30 (95% CI 4.15–20.83), with substantial heterogeneity across studies (I^2^ = 72.30%), indicating moderate-to-high variability in effect estimates (Fig. 3).

**Fig. 4:**
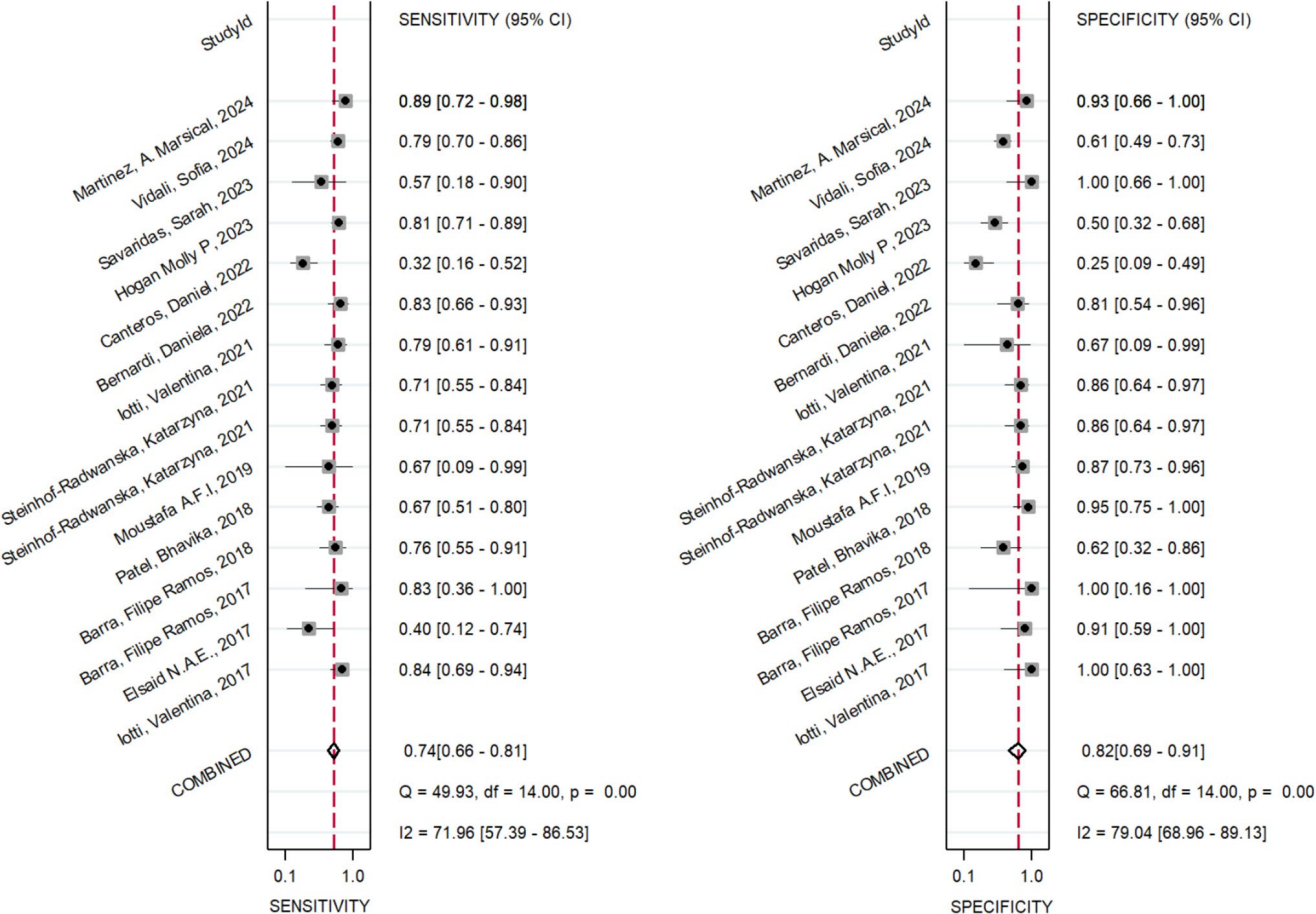
Pooled sensitivity and specificity for contrast-enhanced mammography (CEM) in predicting response to neoadjuvant therapy (NAT) in breast cancer. Circles and lines represent point estimates and 95% confidence intervals, respectively. The diamonds represent the pooled sensitivity and specificity and their 95% confidence intervals

The pooled sensitivity of CEM for detecting pCR was 74% (95% CI 66%–81%; I^2^ = 71.96%), while the pooled specificity was 82% (95% CI 67%–91%; I^2^ = 79.04%) reflecting a moderate-to-high diagnostic performance (Fig. 4).

**Fig. 5:**
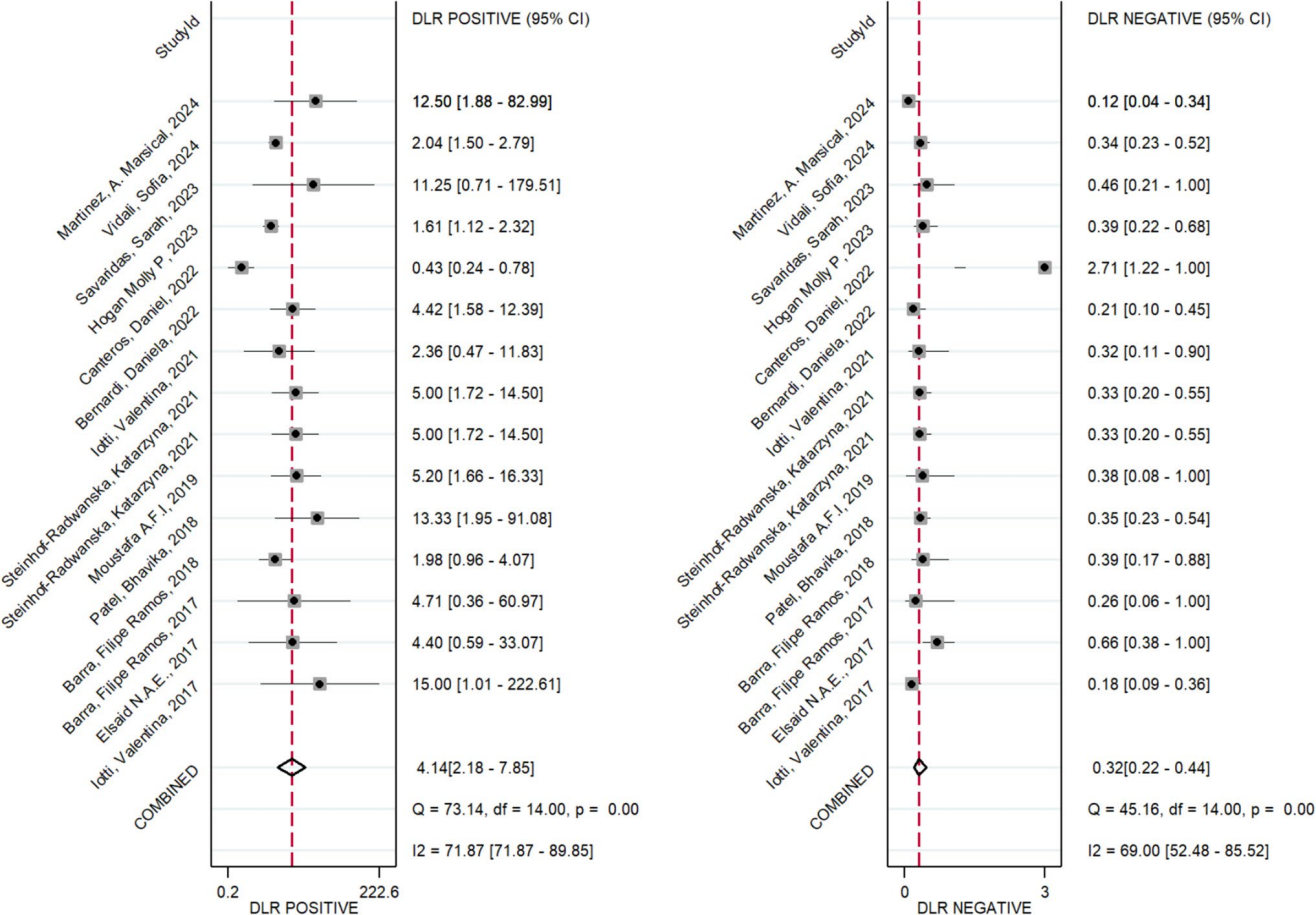
Pooled diagnostic positive likelihood ratio (DLR positive) and negative diagnostic likelihood ratio (DLR negative), for contrast-enhanced mammography (CEM) in predicting response to neoadjuvant therapy (NAT) in breast cancer. Circles and lines represent point estimates and 95% confidence intervals, respectively. The diamonds represent the pooled DLR positive and DLR negative and their 95% confidence intervals

Likelihood ratio analysis showed a LR + of 4.14 (95% CI 2.18–7.85; I^2^ = 71.87%), suggesting that CEM positivity increases the probability of true residual disease, while the LR − of 0.32 (95% CI 0.2420.44; I^2^ = 69.00%) indicates that CEM negativity substantially reduces the likelihood of residual disease (Fig. 5).

**Fig. 6:**
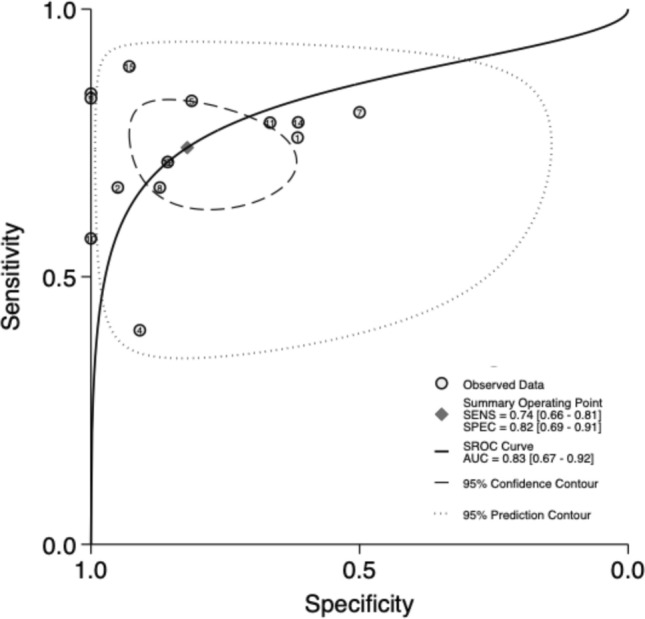
Summary ROC curve with 95% confidence interval for contrast-enhanced mammography (CEM) in predicting response to neoadjuvant therapy (NAT) in breast cancer

The sROC curve (Fig. 6), which plots sensitivity against the false positive rate (1-specificity), yielded an AUC of 0.83 (95% CI 0.67–0.92), supporting CEM’s good overall diagnostic accuracy.

Given a reported pCR prevalence of 31.7%, the PPV was 66%, while the NPV was 87%, further reinforcing CEM’s potential as a reliable imaging modality for evaluating treatment response after NAT.

**Fig. 7:**
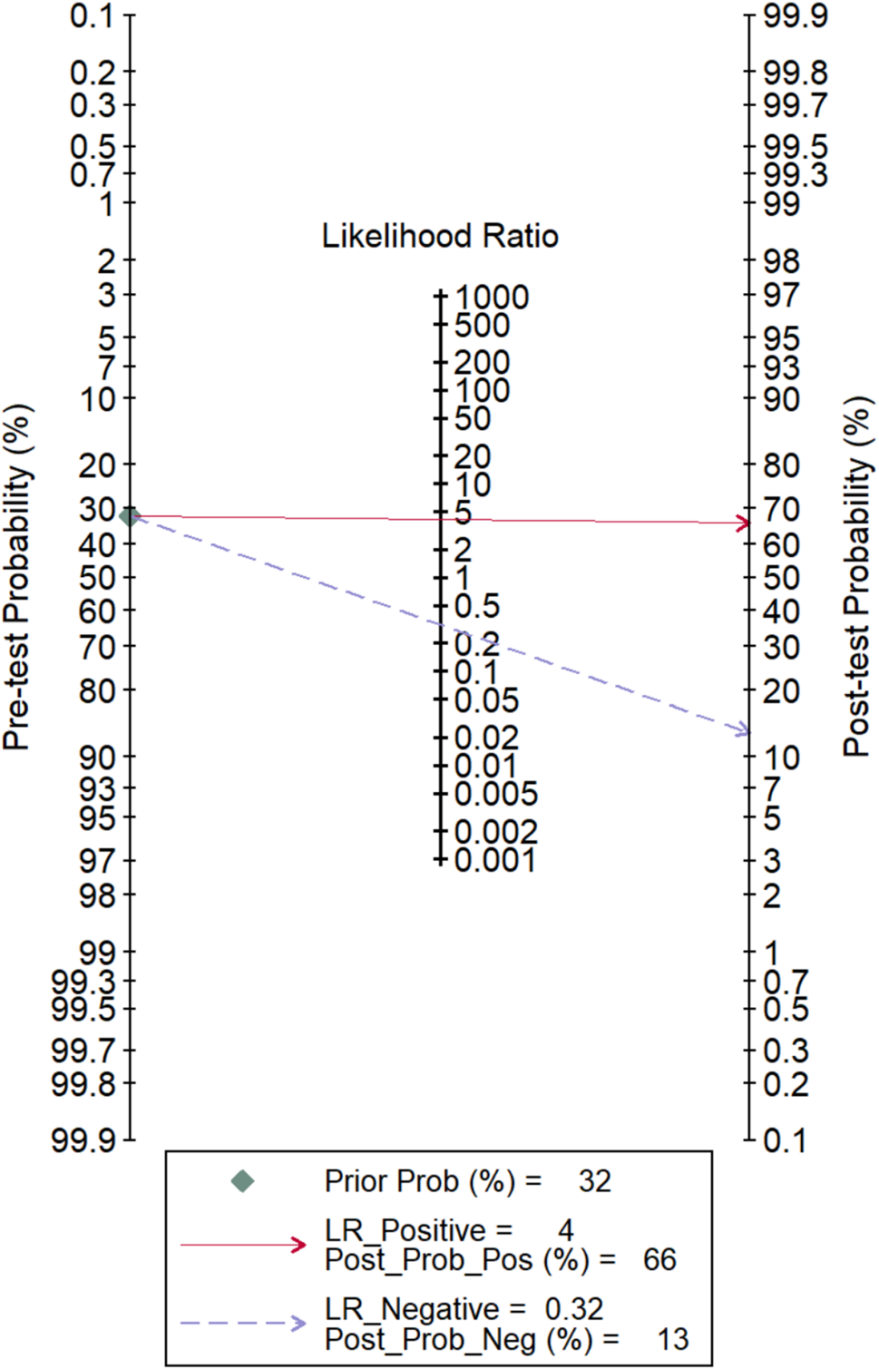
Fagan plot analysis for contrast-enhanced mammography (CEM) in predicting response to neoadjuvant therapy (NAT) in breast cancer

The Fagan nomogram (Fig. 7) indicated a post-test probability of 66% following a positive CEM result and 13% following a negative result, emphasizing the clinical applicability of CEM in NAT response assessment.

**Fig. 8:**
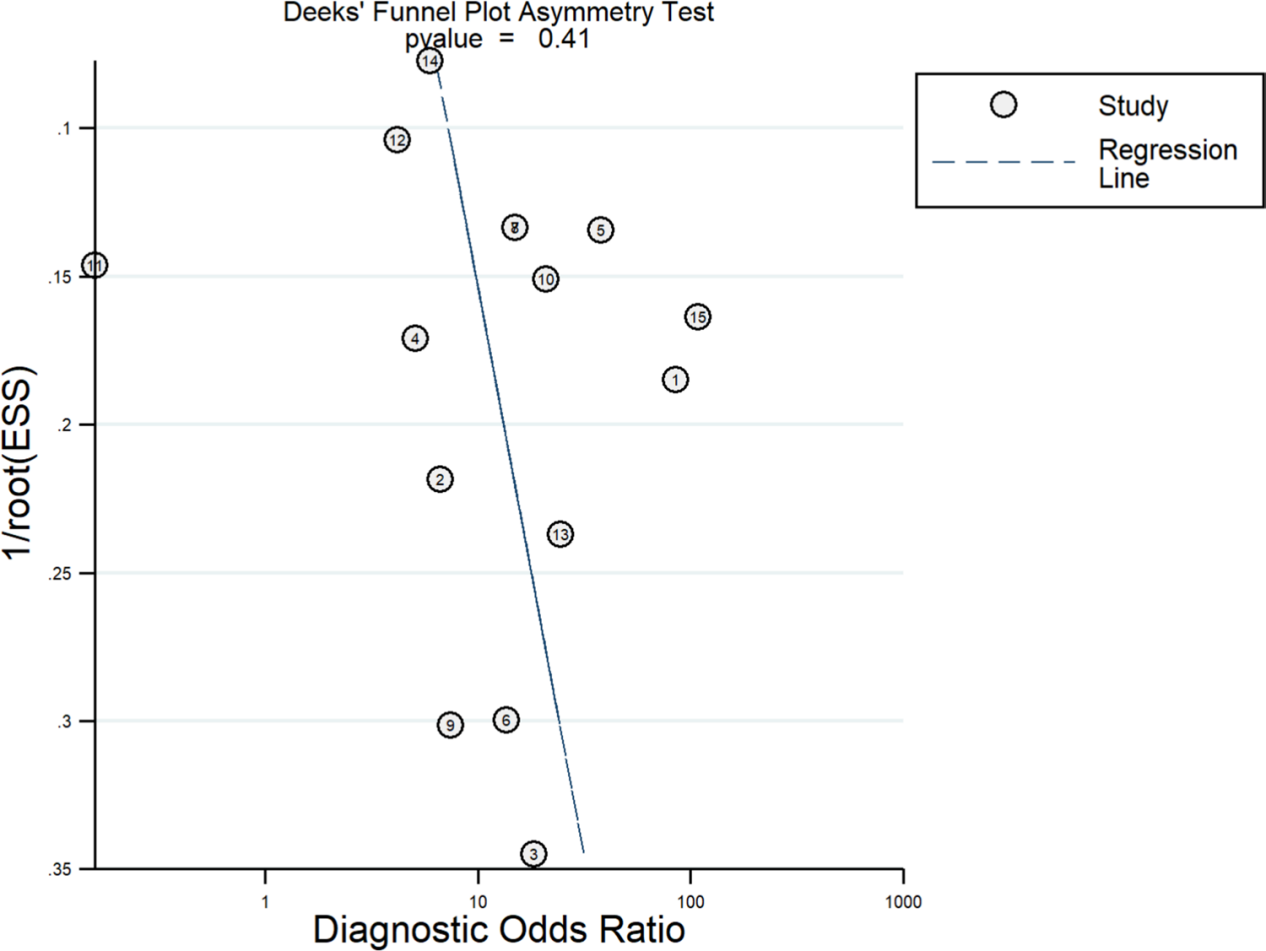
Deek’s funnel plot assessing publication bias

Assessment of publication bias using Deek’s funnel plot (Fig. 8) showed no significant asymmetry (p = 0.409), indicating a low risk of publication bias among the included studies.

### Leave-one-out sensitivity analysis

To assess the impact of individual studies on overall diagnostic accuracy estimates and explore potential sources of heterogeneity, a leave-one-out sensitivity analysis was conducted by sequentially excluding each study from the meta-analysis. The analysis revealed that the removal of the study by Canteros et al. increased DOR to 10.28 (95% CI 6.35–16.65) and reduced heterogeneity to I^2^ = 19.4% (p = 0.242), indicating that this study substantially contributed to the observed variability in pooled estimates.

These findings suggest that while CEM demonstrates consistent diagnostic performance across the included studies, specific methodological differences, patient population characteristics, or imaging protocols in individual studies may influence overall heterogeneity.

## Discussion

This systematic review and meta-analysis evaluated the diagnostic accuracy of CEM in assessing treatment response following NAT, using surgical histopathology as the reference standard. Our findings demonstrated a DOR of 9.30 (95% CI 4.15–20.83), with moderate-to-high heterogeneity (I^2^ = 72.30%). The pooled sensitivity and specificity were 74% (95% CI 66%–81%) and 82% (95% CI 67%–91%), respectively, while the sROC curve yielded an AUC of 0.83 (95% CI 0.67–0.92), indicating good overall diagnostic performance.

The leave-one-out sensitivity analysis identified Canteros et al.'s study as a potential source of heterogeneity, as its exclusion increased the DOR to 10.28 (95% CI 6.35–16.65) and reduced 19.4%. Possible sources of variability introduced by this study include small sample size, differences in pCR definitions, and variation in breast cancer subtype distribution. Prior research suggests that CEM demonstrates higher sensitivity in hormone receptor-negative (HR−) tumors, particularly triple-negative and HER2+ subtypes, due to higher angiogenesis and proliferation rates in these tumor types [33, 37, 38]. Conversely, HR+ tumors tend to exhibit non-mass enhancement or fragmentation into small foci after NAT, potentially leading to underestimation of residual disease [39]. Additionally, the use of taxanes in Canteros et al.'s cohort, which have antiangiogenic properties that reduce microvascular permeability and blood flow, may have contributed to decreased enhancement and reduced CEM sensitivity [33].

The high NPV of 87% indicates that when CEM does not show abnormal enhancement, there is a strong likelihood of true pCR, supporting its use in identifying patients who do not require further surgical intervention. By contrast, the PPV of 66% suggests that CEM alone may not be definitive in confirming pCR, necessitating further histopathologic confirmation. These results highlight the potential role of CEM in personalized treatment planning after NAT: The high NPV supports its use to safely exclude residual disease and guide de-escalation strategies, while the moderate PPV suggests caution in confirming residual disease before treatment intensification.

This finding aligns with existing challenges in post-NAT tumor bed evaluation, which requires precise localization, systematic sampling, and standardized specimen mapping, particularly in patients with negative imaging findings [38]. Furthermore, regional lymph node involvement must be carefully assessed, as isolated residual disease in lymph nodes remains a concern despite the absence of enhancement on CEM [40, 41].

CEM's high NPV makes it an attractive option for integration into post-NAT response assessment protocols, particularly when combined with systematic mapping-guided biopsy techniques. However, a multimodal approach remains essential, ensuring that negative imaging findings are validated by comprehensive tissue sampling [40].

Our findings (sensitivity = 74%, specificity = 82%, AUC = 0.83) are slightly lower than previously reported meta-analyses, which have documented sensitivities of 83–93%, specificities of 68–82%, and AUC values between 0.85 and 0.89 [42, 43]. This discrepancy may be attributed to differences in study selection criteria, patient populations, and NAT regimens. Notably, our study included a broader spectrum of study designs, potentially introducing higher heterogeneity in tumor biology and response patterns. Additionally, variability in pCR definitions among the included studies could have influenced pooled accuracy estimates [42, 43].

When compared to dynamic contrast-enhanced MRI, which has reported sensitivities between 77 and 91% and specificities ranging from 81 to 84%, our findings suggest that CEM performs comparably [42, 44, 45]. While MRI is widely regarded as the gold standard for NAT response assessment, its high cost, longer examination times, and lower patient tolerance limit its accessibility [46]. Additionally, patients undergoing MRI often require additional mammography post-NAT, whereas CEM provides both anatomical and functional imaging in a single session, offering a practical and cost-effective alternative [47, 48].

CEM combines low-energy imaging (LEI) for detailed anatomical visualization and recombined imaging (RI) for functional enhancement assessment. Previous studies indicate that incorporating LEI improves sensitivity, particularly in detecting residual disease, though at the expense of lower specificity [34]. This trade-off may stem from LEI’s ability to detect microcalcifications, which can be missed on MRI, leading to false negatives, while also contributing to false positives [34]. Furthermore, inflammatory changes post-NAT, as well as reduced vascularity due to necrosis or fibrosis, can lead to underestimation of residual disease, further complicating interpretation [49].

Balancing sensitivity and specificity remains crucial to optimizing CEM performance. Standardizing contrast injection protocols, imaging acquisition techniques, and interpretation criteria could help reduce variability across studies and enhance diagnostic confidence in clinical practice.

Despite an I^2^ value below 50% after sensitivity analysis, suggesting low residual heterogeneity, we acknowledge that the initial heterogeneity was substantial (I^2^ = 72.30%). Exploratory meta-regressions assessing publication year and sample size did not reveal significant associations with the diagnostic odds ratio and did not meaningfully reduce between-study variance. Subgroup analyses based on tumor subtypes, NAT regimens, or CEM protocols were not feasible due to limited data reporting. Further subgroup analyses, as well as patient-level meta-analyses and data pooling from high-volume centers, are warranted to provide deeper insights and address current limitations. Variability in NAT protocols across studies may also have influenced response assessment. Therefore, well-designed, large-scale prospective studies with standardized imaging and histopathologic criteria are needed to confirm CEM’s role in post-NAT response evaluation.

In the included studies, pathological complete response (pCR) was generally defined as ypT0/is N0, but some variability was observed regarding the inclusion of cases with residual in situ disease (pTis). This distinction has recognized clinical relevance; however, due to inconsistent reporting of pCR definitions across studies, it was not feasible to perform a separate analysis comparing pT0 versus pTis. These differences may have contributed to part of the observed heterogeneity and should be addressed through standardized pCR criteria in future research.

## Conclusion

Our meta-analysis confirms that CEM demonstrates good diagnostic performance in assessing treatment response after NAT, with an AUC of 0.83, high NPV, and diagnostic accuracy comparable to MRI. These findings reinforce CEM’s potential role as a valuable imaging modality in post-NAT response assessment, particularly given its ability to provide both anatomical and functional information in a single, cost-effective, and widely available examination.

Compared to dynamic contrast-enhanced MRI, which remains the gold standard for NAT response evaluation, CEM offers several advantages, including shorter acquisition times, reduced costs, and improved patient tolerance. Additionally, CEM has demonstrated high inter-reader consistency, making it a promising tool in centers with varying levels of radiological expertise. However, challenges remain in optimizing the balance between sensitivity and specificity, particularly in distinguishing true residual disease from post-treatment changes, such as fibrosis or inflammatory enhancement.

Despite its strengths, further high-quality, prospective, multicenter studies are required to standardize imaging acquisition protocols, refine interpretation criteria, and evaluate CEM’s performance across different tumor subtypes and NAT regimens. Future research should also explore integrating CEM into multimodal post-NAT strategies, potentially in combination with systematic biopsy mapping techniques, to enhance diagnostic accuracy and improve patient stratification for tailored surgical management.

While our findings support CEM as a reliable imaging option for post-NAT assessment, particularly in scenarios where MRI is unavailable or contraindicated, its clinical implementation should be guided by ongoing validation studies to ensure optimal integration into evidence-based breast cancer management protocols.

## Abbreviations

CEM: Contrast-enhanced mammography
NAT: Neoadjuvant therapy
pCR: Pathological complete response
DOR: Diagnostic odds ratio
sROC: Summary receiver operating characteristic
MRI: Magnetic resonance imaging
QUADAS-2: Quality assessment of diagnostic accuracy studies-2
